# IDO1 involvement in mTOR pathway: a molecular mechanism of resistance to mTOR targeting in medulloblastoma

**DOI:** 10.18632/oncotarget.9284

**Published:** 2016-05-11

**Authors:** Valentina Folgiero, Evelina Miele, Andrea Carai, Elisabetta Ferretti, Vincenzo Alfano, Agnese Po, Valentina Bertaina, Bianca Maria Goffredo, Maria Chiara Benedetti, Francesca Diomedi Camassei, Antonella Cacchione, Franco Locatelli, Angela Mastronuzzi

**Affiliations:** ^1^ Department of Hematology/Oncology and Stem Cell Transplantation, Bambino Gesù Children's Hospital, IRCCS, Rome, Italy; ^2^ Department of Molecular Medicine, Sapienza University, Rome, Italy; ^3^ Center for Life NanoScience@Sapienza, Istituto Italiano di Tecnologia, Rome, Italy; ^4^ Department of Neuroscience and Neurorehabilitation, Neurosurgery Unit, Bambino Gesù Children's Hospital, IRCCS, Rome, Italy; ^5^ Department of Laboratory Medicine, Bambino Gesù Children's Hospital, IRCCS, Rome, Italy; ^6^ Department of Laboratories, Pathology Unit, Bambino Gesù Children's Hospital, IRCCS, Rome, Italy; ^7^ Department of Pediatric Science, University of Pavia, Pavia, Italy

**Keywords:** mTOR, IDO1, Treg, MB, CCL2

## Abstract

Medulloblastoma (MB) is the most common malignant brain tumor in children. Despite therapeutic advancements, high-risk groups still present significant mortality. A deeper knowledge of the signaling pathways contributing to MB formation and aggressiveness would help develop new successful therapies. The target of rapamycin, mTOR signaling, is known to be involved in MB and is already targetable in the clinical setting. Furthermore, mTOR is a master metabolic regulator able to control cell growth versus autophagy decisions in conditions of amino-acid deprivation that can be due to IDO1 enzymatic activity. IDO1 has been also implicated in the regulation of inflammation, as well as of T cell-mediated immune responses, in a variety of pathological conditions, including brain tumors. In particular, IDO1 induces expansion of regulatory T-cells (Treg), preventing immune response against tumor cells. Analysis of 27 MB tissue specimens for the expression of both mTOR and IDO1 showed their widespread expression in all samples. Testing their cooperation *in vitro*, a significant involvement of IDO1 in mTOR immunogenic pathway was found, able to counteract the aim of rapamycin treatment. In MB cell lines, inhibition of mTOR strongly induced IDO1 expression and activity, corroborating its ability to recruit Treg cells in the tumor microenvironment. The mTOR/IDO1 cross talk was found to be strictly specific of MB cells. We demonstrated that mTOR pathway cross talks with IDO1 pathway to promote MB immune escape, possibly contributing to failure of mTOR- targeted therapy.

## INTRODUCTION

Medulloblastoma (MB) is the most common malignant brain tumor in children, with several pathways implicated in its development [1, 2]. MB belongs to the embryonic tumor family and recent genomic studies have led to a novel classification into four molecular subgroups (i.e. WNT; Sonic Hedgehog Homolog, SHH; Group 3 and Group 4). WNT and SHH groups are characterized by activation of WNT/beta-catenin and SHH pathway, respectively. Group 3 and 4 are less genetically characterized, the first predominantly showing C-MYC amplification while Group 4 is often characterized by MYCN amplification and presence of iso-chromosome 17q [3, 4]. Current treatments can cure about 80% of MB patients, however at the cost of significant long-term toxicities [5]. Preclinical studies have revealed the potential of molecular targeted therapy in treating MBs [6]. The PI3K/Akt/mTOR pathway is a key player in many types of cancer and its pharmacological targeting *via* molecular inhibitors is proving to be promising in MB as well [7]. In particular, mTOR is a member of the PI-3 kinase-like kinase family (PIKKs), which plays an integral role in coordinating cell growth and division in response to growth factors, nutrients and energy status of the cell [8]. Upon activation, mTOR is phosphorylated on several residues, and such phosphorilated form (P-mTOR) is involved in development and progression of MB [9, 10]. Indeed, a recent study in 40 primary MB specimens suggested a possible synergistic effect between mTOR inhibitors and conventional chemotherapy in MB with activated mTOR pathway [11]. Furthermore, mTOR is a master metabolic regulator, with a key role as a nutrient sensor being able to control cell growth versus autophagy decisions in conditions of amino acid deprivation [12]. In particular, Tryptophan (TRP) depletion is governed by indoleamine 2,3-dioxygenase (IDO1), which catalyzes the first rate-limiting step in the consumption of TRP [13]. Such IDO1-mediated TRP depletion from tissues alters inflammation, as well as T-cell mediated immune response, in a variety of pathological settings, including cancer and, in particular, brain tumors [14]. Interferon gamma (IFNγ), a potent IDO1 inducer, depletes TRP and represses mTOR activity in Hela cells [15]. In glioblastoma, the IDO1-mediated response depends on the increase of chemokines like CCL2, able to attract Tregs [16]. More in general, IDO1-mediated TRP depletion establishes an immunosuppressive environment, amplifying tolerogenic antigen-presenting cells (APC), expanding Treg and downregulating cytotoxic T-cell activity, thus preventing immune response to tumor cells [17]. Based on this body of evidence, the aim of our study was to evaluate the IDO1/mTOR pathway crosstalk in MB and its role in immune escape.

## RESULTS

### mTOR and IDO1 are co-expressed in MB tissue samples

Histological analysis in our population of 27 MB patients showed 15 classic, 7 desmoplastic and 5 anaplastic forms. Based on age the patients were divided in infants (6 children under age of 3) and child (21 patients). We found a predominance of molecular subgroups 3 and 4 in our series; WNT = 6, SHH = 5, group 3 = 9 and group 4 = 7 patients (Table 1).

**Table 1:** Clinical and pathological characteristics of patients

Patients	Age	Histopathology	mTOR	P-mTOR	IDO1	Mol. subgroup
#1	Child	Anaplastic	3+	2+	3+	3
#2	Child	Classic	3+	2+	2+	4
#3	Child	Desmoplastic	3+	2+	2+	SHH
#4	Child	Classic	3+	1+	3+	WNT
#5	Child	Classic	3+	1+	1+	4
#6	Infant	Classic	3+	0	2+	WNT
#7	Infant	Classic	1 +	1+	1+	3
#8	Child	Classic	2+	1+	2+	SHH
#9	Child	Desmoplastic	1 +	0	2+	4
#10	Child	Classic	2+	1+	3+	4
#11	Child	Anaplastic	3+	1+	3+	3
#12	Child	Classic	2+	1+	3+	WNT
#13	Infant	Anaplastic	3+	0	3+	3
#14	Child	Anaplastic	2+	1+	3+	3
#15	Infant	Desmoplastic	2+	1+	3+	SHH
#16	Child	Classic	2+	2+	1+	3
#17	Child	Desmoplastic	3+	1+	1+	SHH
#18	Child	Classic	3+	2+	2+	4
#19	Child	Classic	3+	1+	2+	WNT
#20	Infant	Desmoplastic	3+	1+	1+	SHH
#21	Child	Anaplastic	3+	2+	2+	3
#22	Child	Desmoplastic	3+	2+	1+	4
#23	Child	Classic	2+	1+	1+	4
#24	Child	Classic	2+	1+	2+	WNT
#25	Child	Classic	3+	1+	3+	WNT
#26	Infant	Classic	3+	0	2+	3
#27	Child	Desmoplastic	3+	2+	3+	3

**Figure 1:**
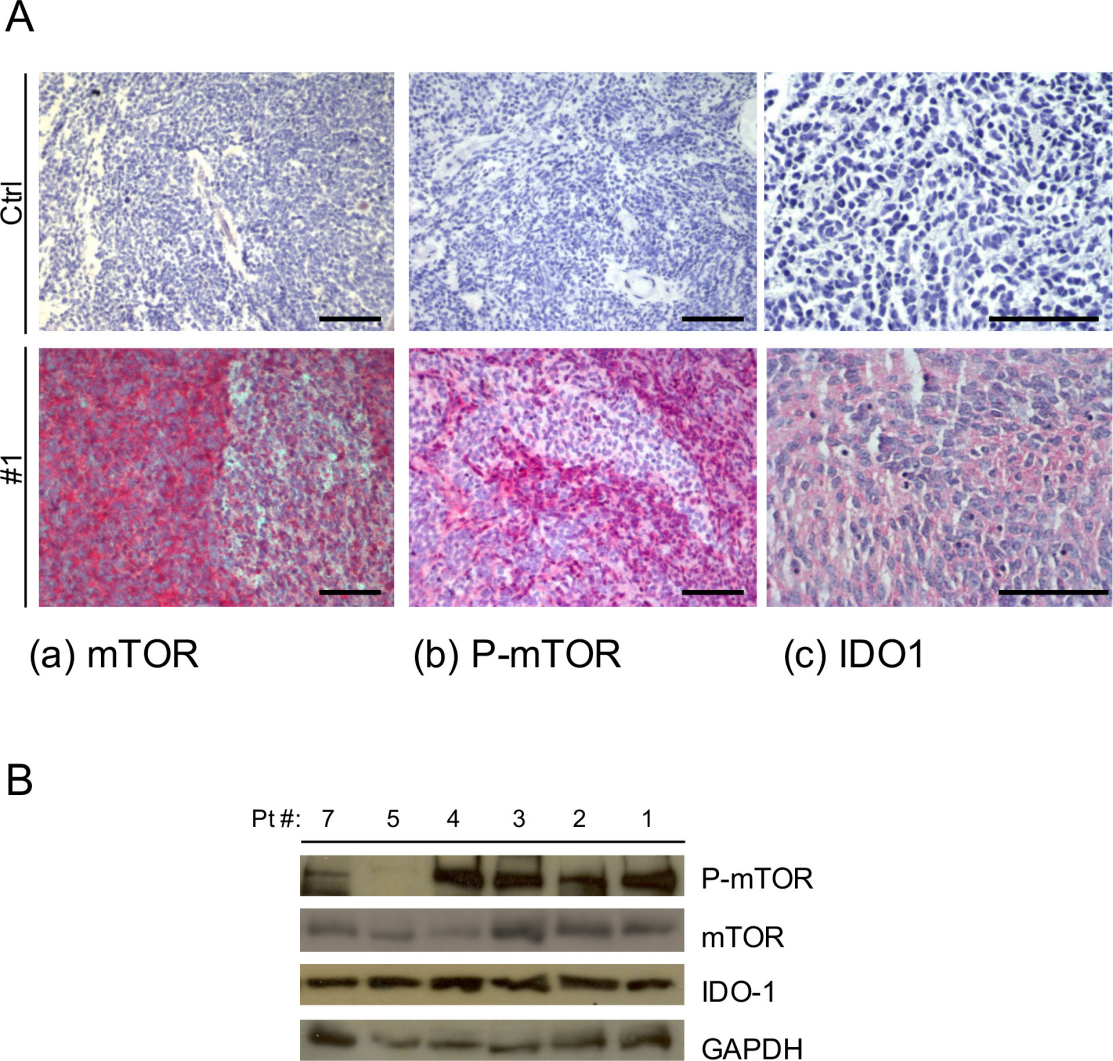
**(A)** Representative IHC staining of human MB samples of (a) mTOR 20X, (b) P-mTOR 20X and (c) IDO1 40X and negative control for each antibody (CTRL) Scale bar 100 μm. **(B)** Representative WB analysis performed on MB tissue samples for the expression of P-mTOR, mTOR and IDO1. GAPDH Ab expression was used as loading control.

Immunohistochemistry (IHC) analysis on surgical samples revealed that all were positive for mTOR and IDO1 and that 85% expressed P-mTOR, compared with negative controls (Figure 1A, Table 1), irrespective of molecular subgroup. Similarly, looking at IDO1 mRNA expression in available microarray datasets (R2 and Oncomine) we did not find changes across MB subgroups (Supplementary Figure 1). Western blot analysis confirmed the expression pattern, documenting the presence of IDO1 in MB tissue (Figure 1B).

### mTOR inhibition induces IDO1 expression in MB cell line

**Figure 2:**
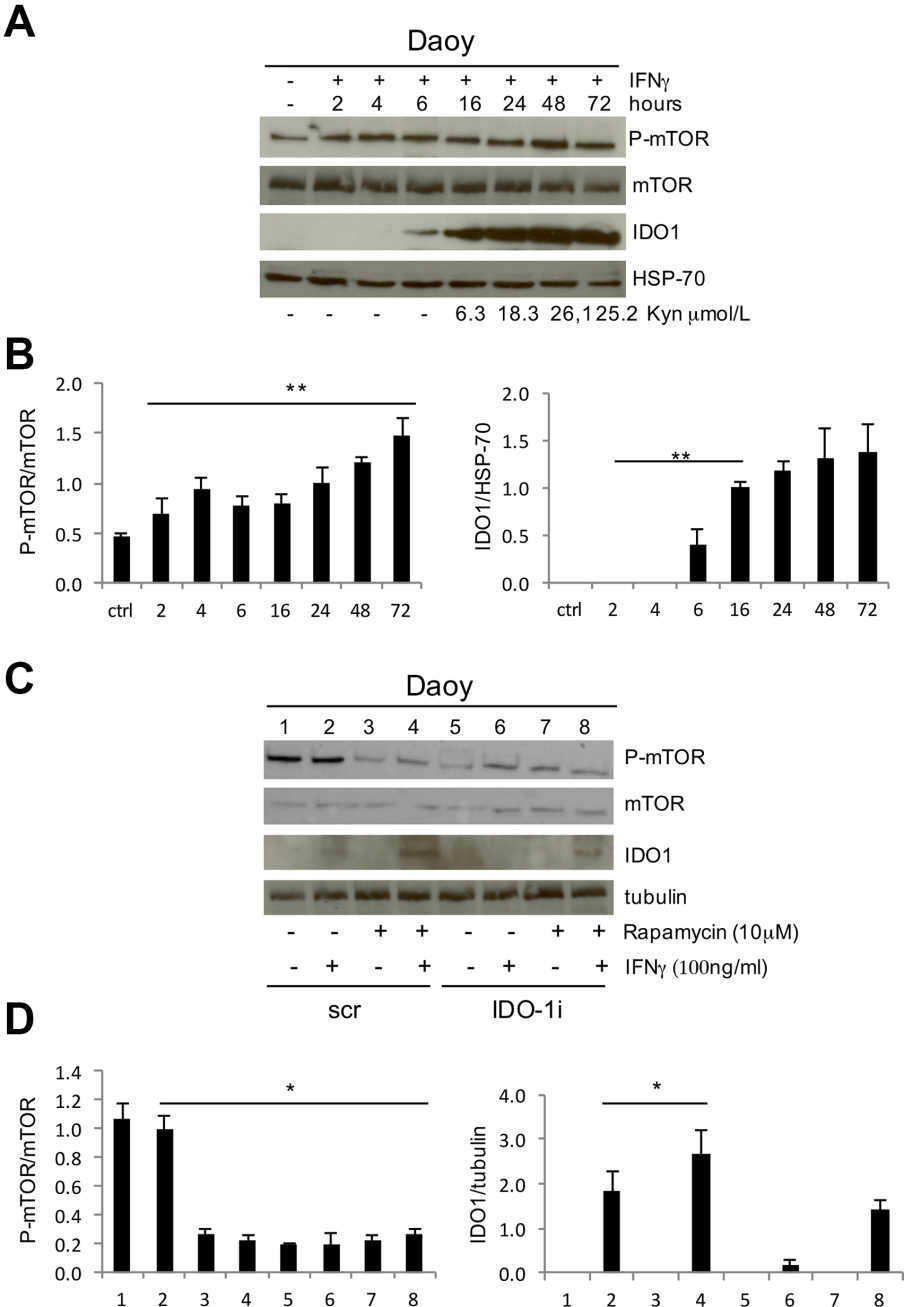
**(A)** IFNγ stimulation of DAOY cell line at subsequent time points. P-mTOR, mTOR and IDO1 expression was evaluated by WB analysis and compared with kynurenine release in the culture supernatants. HSP70 Ab was used as loading control. (**B**) Graphic representation of densitometric analysis of three independent experiments. P-mTOR was normalized based on the values of mTOR expression (left), IDO1 on HSP-70 expression (right) (**P ≤ 0.01). (**C**) Rapamycin treatment of DAOY cell line in combination with IFNγ stimulation upon IDO1 interference. P-mTOR, mTOR and IDO1 modulations were analyzed by WB. Tubulin Ab was used as loading control. (**D**) Graphic representation of densitometric analysis of three independent experiments. P-mTOR was normalized based on the values of mTOR expression (left), IDO1 on tubulin expression (right) (*P ≤ 0.05). Densitometry was performed with Image J program.

In DAOY cells, IDO1 was expressed only after IFNγ stimulation. In particular, IDO1 was induced 6 hours after IFNγ treatment, while IDO1 enzymatic activity, measured as the amount of the metabolite kynurenine detectable in the supernatant, started after 16 hours (Figure 2A, 2B). IDO1 enzymatic activity reached its peak after 48 hours and correlated with the highest levels of mTOR phosphorylation status, suggesting a functional correlation between them. To better understand mTOR-signaling-dependent modulation of IDO1 expression and function, we added the mTOR inhibitor rapamycin to DAOY cells followed by IFNγ stimulation. Unexpectedly, we observed that P-mTOR downregulation strongly enhanced the IFNγ-dependent IDO1 expression compared to control samples (Figure 2C, 2D). Furthermore, induction of IFNγ-dependent IDO1 expression by rapamycin treatment was so effective to revert IDO1 interference when compared with that induced by IFNγ alone (Figure 2C, 2D). Altogether, these findings suggest a crosstalk between the two molecules, and that downregulation of mTOR strongly contributes to IFNγ-mediated IDO1 protein overexpression. In addition, IDO1 interference negatively affected mTOR phosphorylation, suggesting a functional loop deserving further investigation.

### Rapamycin-dependent IDO1 expression induces Treg expansion

**Figure 3:**
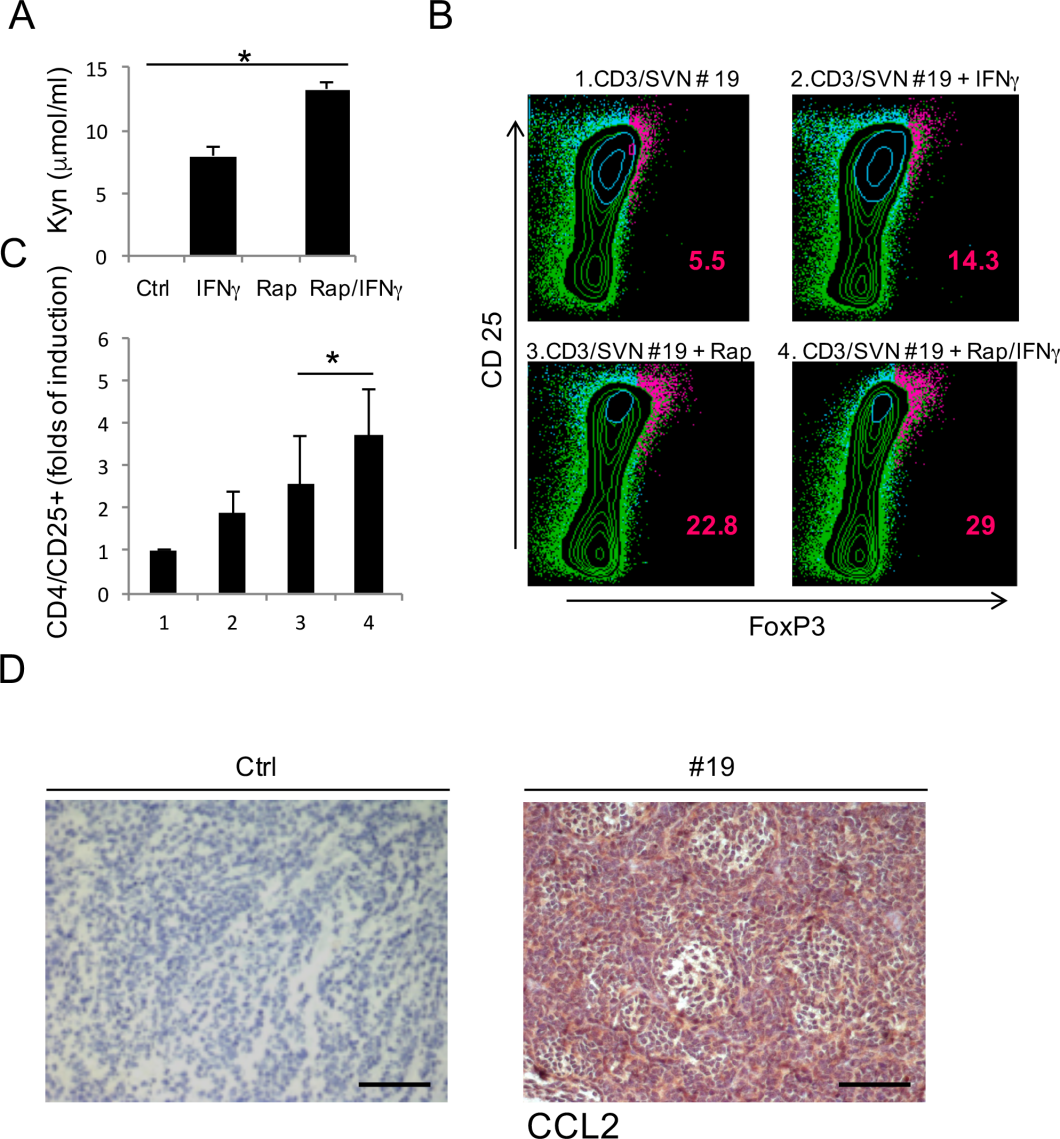
**(A)** Evaluation of kynurenines released in the supernatants of primary cell lines obtained from three different MB tissue samples, in the specific culture conditions. Bars are representative of mean values and standard deviation recorded in three independent experiments (*P ≤ 0.05). (**B**) Representative CD4/CD25/FoxP3 staining in CD3+ cells stimulated with supernatant obtained from specific cultures (1–4) of primary MB cell line (Patient #19), to evaluate the percentage of induced Treg cells. The percentage of cells staining positively is indicated. (**C**) Graphic representation of CD25/FoxP3+ cells fold of induction produced by three independent experiment (*P ≤ 0.05). (**D**) Representative CCL2 chemokine IHC staining (20×) of human MB samples compared with negative control (#19) Scale bar 100 μm.

IDO1 contributes to the immune escape mechanism inducing the expansion of Treg cells through its catabolites kynurenines, that are detectable in the culture supernatants (SVN) as shown in Figure 3A. For this reason, we evaluated the effect of mTOR inhibition on IDO1 immunosuppressive activity by analyzing the expansion of Treg cells, identified by the concomitant CD4/CD25/FOXP3+ expression on peripheral lymphocyte population. CD3+ cells were magnetically sorted from peripheral blood of a healthy donor and cultured for 5 days in SVN from primary MB cell cultures grown under different conditions. In Figure 3B and 3C, we show that the SVN obtained from MB cells stimulated with IFNγ was able to expand Treg cells population more effectively when compared to untreated sample. This was due to the kynurenine released into the SVN by IDO1 induction as previously demonstrated [18] (Figure 3A). SVN obtained from MB cells treated with rapamycin favored upregulation of FoxP3 expression [19]. In particular, we observed a 22.8% of Treg cells with rapamycin-MB-SVN and even a higher amount or Treg cells reaching 29% when SVN obtained from MB cells treated with rapamycin and IFNy was employed, this latter value doubling the one observed with IFNy alone (Figure 3B). This finding suggests that IDO1 expression and activity induced by rapamycin constitute a stronger IDO1-dependent immune escape mechanism. A possible mediator of IDO1-dependent Treg expansion is the increase of chemokines like CCL2 which attracts Treg cells, leading to a global tumor immune tolerance [16, 20]. Thus, we evaluated the level of CCL2 expression in MB tissue samples by IHC compared with negative control (Figure 3D). As shown in Table 2, the totality of samples were highly positive for the expression of CCL2, supporting our hypothesis that IDO1 expression, by recruiting Treg cells, could create an immune-tolerance microenvironment. Such mechanism could be responsible for failure of mTOR therapy in MB.

**Table 2:** CCL2 IHC analysis

Patients	CCL2
#1	2+
#2	2+
#3	3+
#4	2+
#5	3+
#6	3+
#7	2+
#8	2+
#9	1+
#10	2+
#11	2+
#12	1+
#13	2+
#14	2+
#15	2+
#16	3+
#17	1+
#18	2+
#19	3+
#20	2+
#21	3+
#22	2+
#23	2+
#24	3+
#25	2+
#26	2+
#27	3+

### mTOR-IDO1 interaction is context-dependent in brain tumors

**Figure 4:**
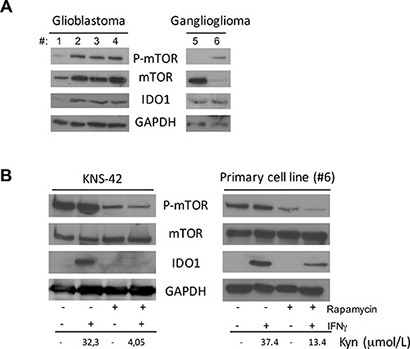
**(A)** Analysis of P-mTOR, mTOR and IDO1 in 4 Glioblastoma tissue samples (left panel), and 2 Ganglioglioma samples (right panel) by WB. GAPDH was used as loading control. (**B**) Rapamycin treatment in combination with IFNγ stimulation in KNS-42 cell line (left panel) and Ganglioglioma primary cell line from patient #6 (right panel). P-mTOR, mTOR and IDO1 modulation was analyzed by WB. GAPDH Ab was used as loading control. IDO1 expression was correlated with kynurenine release in culture supernatants. Similar results were obtained in 3 independent experiments.

To verify whether this peculiar crosstalk between mTOR and IDO1 observed in DAOY cells could be extended also to other high-grade or low-grade brain tumors, we evaluated the expression of the two molecules by WB on 4 glioblastoma and 2 ganglioglioma tissue specimens. All samples were positive for IDO1, p-mTOR and mTOR (Figure 4A). Afterwards, we used the glioblastoma cell line KNS-42 and a primary cell line obtained from a ganglioglioma tissue sample (#6). As shown in Figure 4B, IFNγ-mediated induction of IDO1 was strongly reduced by mTOR inhibition with rapamycin suggesting that functional relationship between mTOR and IDO1 might be specific of MB among CNS tumors.

## DISCUSSION

Recent advances in identifying molecular pathways that enhance amino acid catabolism and downstream mechanisms that affect immune cells response support the notion that it can regulate innate and adaptive immune cells in pathological settings. Limited access to amino acids during immune cell activation may compromise immune responses by inhibiting immune cell division, differentiation, maturation, migration, and acquisition of new effector functions [12]. In particular, TRP catabolism has emerged as a powerful mechanism of peripheral immune tolerance, contributing to maintain homeostasis by preventing autoimmunity or immunopathology that would result from uncontrolled and overacting immune response. IDO1 is one of three enzymes able to catalyze TRP degradation along the kynurenine pathway [21]. As a result, TRP is locally depleted while TRP catabolites, including kynurenine and its derivatives, accumulate. Tumors divert the immunosuppressive IDO1 function to their own benefit in their continue efforts to resist immune aggression [22]. A number of human tumors are known to express IDO1 in a constitutive manner and most start expressing IDO1 when exposed to IFNγ [14]. Ectopic expression of IFNγ during CNS development in mice results in cerebellar tumors that share the major molecular and pathological characteristics of human MB [23]. Among cell signaling pathways possibly affected by IDO1, mTOR has a key role due to its capacity of being a nutrient sensor influenced by amino acid levels [17]. It has been demonstrated that TRP deprivation triggers autophagy in an mTOR-dependent manner and induction of autophagy is reversed by restoring essential amino acids like TRP. In addition, mTOR acts as a pivotal immune modulator playing a crucial role in antigen responsiveness in CD4^+^ cells [24]. This effect seems to be mediated by an influence of mTOR inhibition on naturally occurring regulatory Treg cells, which have a role in immunological tolerance. MB formation and growth has been associated with multiple molecular aberrations, including those of the mTOR pathway [25]. Our findings showed that 100% of MB tissue samples are positive for mTOR and IDO1, and that 85% of them express P-mTOR. In addition, we found their expression to be independent from MB molecular subgroup. However such lack of correlation of IDO1 expression with the molecular subgroups could be due to the few number of MB analyzed and the lacking of available data-set analysis on protein expression in MB. Intriguingly, rapamycin was able to induce IDO1 expression, contributing to tumor immune escape mechanism. Since IDO1 interference affected mTOR phosphorylation, we propose IDO1 as a possible molecular target in MB. Indeed, this IDO1-centered concept is supported by numerous preclinical studies in models of tumor immunity [26]. Furthermore, it is known that prolonged administration of an mTOR inhibitor, rapamycin, results in a pronounced increase in number of Treg [27]. Given the critical role of Treg cells in immune tolerance, it is important to delineate signaling pathways that either positively or negatively regulate their differentiation. The mechanism by which mTOR inhibition enhanced FoxP3 expression has not been clarified yet, but it is known that mTOR could act directly on signal transducer and activator of transcription (STAT3), known to be involved in FoxP3 increased transcription [28, 29]. Since we previously demonstrated that IDO1 induction is dependent by STAT3 phosphorylation [24], we hypothesize that IDO1 be a possible candidate for mTOR-dependent Treg expansion. Indeed, in a primary MB cell line, we demonstrated that induction of IDO1 after rapamycin treatment strongly promote the recruitment of Treg cells. This hypothesis is supported by the high expression of the Treg attractive chemokine, CCL2, observed by IHC in all human MB samples. Since IDO1 inhibitors are now being developed clinically for the treatment of several cancers with the aim of reversing cancer-associated immune suppression [30], we speculate that a possible targeting of IDO1 in MB could improve tumor eradication by acting at different levels. Indeed, IDO1 down-regulation can impair both mTOR activation and Treg-dependent tumor immune escape mechanism. Furthermore, we found this effect to be specific for MB, since it was not found in different brain tumors and we provide evidence of molecular crosstalk between mTOR and IDO1. The unexpected Treg-mediated immune-escape response induced by rapamycin treatment suggests caution in using mTOR inhibitors in MB, at least as single agent therapy. Advancements in molecular profiling might allow implementation of MB subgrouping with IDO1 activation profiling, indicating possible benefit from molecularly driven combinatorial approaches, including IDO1 targeted therapy.

## MATERIALS AND METHODS

### Human tissue samples

Formalin-fixed, paraffin-embedded samples of primary MB from 27 consecutive patients treated at Bambino Gesù Children's Hospital between 2013 and 2015 were examined. A part of every sample was cryopreserved for protein extraction at time of surgery. MB diagnosis was made complying with WHO 2007 criteria. The study was approved by the Institutional Review Board. MB molecular subgroup was determined by qRT-PCR as previously described [31, 32].

### Gene expression data set analyses

The Oncomine platform (https://www.oncomine.org/resource/login.html) and R2: Genomics Analysis and Visualization Platform (http://r2.amc.nl) were used to interrogate all data sets. Two data sets in R2 ([33], RNA) and [34] DNA) and Three in Oncomine ([20] RNA and [35], for both RNA and DNA) were interrogated to assess IDO1 levels across MB subgroups.

### Immunohistochemistry

Three mmm paraffin-embedded sections were dehydrated, pretreated with avidin block for 15 min and biotin block for 15 min, then incubated with bovine serum albumin (BSA) for 30 min and overnight at 4°C with monoclonal rabbit antibodies against mTOR (Cell Signaling Technology, 1:50 dilution, PT-link pre-treatment at high pH) and Phospho-mTOR (Cell Signaling Technology, 1:100 dilution, PT-link antigen retrieval at high pH). Incubation with biotinylated secondary antibodies for 15 min at room temperature and incubation with alkaline phosphatase conjugated streptavidin for 15 min at room temperature were performed. Other 3 mmm paraffin embedded sections were incubated with primary antibodies against IDO1 (Santa Cruz Biotechnology, rat monoclonal Ab, 1:250) for 60 min at 37°C and then with secondary antibodies (polyclonal rabbit anti-rat biotynilated Igs Dako, 1:200) for 30 min at 37°C. Chromogenic development was obtained using Fast Red (Dako Real kit). Other 3 mmm paraffin-embedded sections were incubated with primary antibodies against CCL2 (R&D Systems, monoclonal mouse, 1:25 dilution, PT-link antigen retrieval at high pH) over night at 4°C and then with Flex HRP (enzyme horseradish peroxidase, Dako) for 20 min at room temperature. Chromogenic development was obtained using 3,3′-diaminobenzidine tetrahydrochloride with 0.03% hydrogen peroxidase (Dako). Finally, all sections were counterstained with haematoxylin, cleared and mounted. Sections were visualized with a Zeiss AXIO microscope and digital image acquisition performed with attached Zeiss AxioCam MRc5 using the AxioVision 4.8 software.

### Cell cultures and treatments

Daoy MB cell line was purchased from ATCC (HTB-186) and maintained in RPMI medium supplemented with 10% fetal calf serum (FCS) (Gibco), L-glutamine and penicillin/streptomycin (Euroclone) at 37°C in humidified 5% CO_2_ air. KNS-42 high-grade glioma cell line was obtained from Japan Health Sciences Foundation, Health Science Research Resources Bank and grown in DMEM/F12 medium + 10% FCS at 5% CO_2_, as previously described [36]. MB and ganglioglioma primary cell lines were obtained from fresh tissue samples and maintained in culture with RPMI supplemented with 20% FCS. After 1 week, the supernatant was removed from cultures and replaced with fresh medium. Two weeks from the start of the culture, cells were harvested and replated to perform the experiments. In order to study IDO1/mTOR crosstalk, cells were plated at 1.6 × 10^5^ cells/well in 6-wells plates. On day two, medium was replaced with a serum-free one for rapamycin (Cell Signaling) addition (10 mM) and maintained overnight. On day three, IFNγ (Immunological Sciences) was added at 100 ng/ml fresh medium for 16 hours. At the end of treatment, supernatant was collected for the quantification of kynurenine production and the cells were lysed for protein extraction.

### IDO1 interference

Daoy cells were plated at 1.6 × 10^5^ cells/well in 6-wells plates. The day after, cells were transiently transfected by Lipofectamine 2000 (ThermoFisher) with control shRNA or IDO shRNA plasmid (Santa Cruz Biotechnology) following the manufacturer's protocol. Twenty four hours after transfection cells were stimulated with IFNγ for 16 hours. At the end of treatment, supernatants were collected for the quantification of kynurenine production and cells were lysed for protein extraction.

### Western blotting

At the end of treatments, cell pellets were lysed with RIPA buffer [150 mM NaCl, 1% NP-40, 0.5% sodium deoxycholate, 0.1% SDS, 50 mM Tris–HCl (pH = 8), 1 mM PMSF, 1 mM EGTA, 50 mM NaF, 50 mM Na_3_VO_4_ and protease inhibitors (Roche, Milan, Italy)]. Cell lysates were incubated on ice for 20 minutes and clarified by centrifugation at 14,000 rpm for 20 minutes. Cell extracts obtained with RIPA buffer were boiled for 5 minutes at 95°C and analyzed by SDS-PAGE. Samples were transferred onto nitrocellulose membrane (Bio- Rad, Milan, Italy). Blots were probed with primary antibodies (anti-P-mTOR, mTOR, IDO1, GAPDH from Cell Signaling; anti-actin and anti-tubulin from Santa Cruz Biotechnologies) washed and developed with HRP- conjugated rabbit or mouse secondary antibodies (Cell Signaling), as appropriate.

### IDO1 activity

TRP and kynurenine levels were measured with reverse-phase HPLC Agilent Technologies 1200. Briefly, sample aliquots (200 μL) were diluted with 200 μL potassium phosphate buffer (0.05 mol/L pH 6.0) containing the internal standard 3-nitro-L-tyrosine (100 mmol/L). Proteins were precipitated with 50 μL of trichloroacetic acid (2 mol/L). Capped tubes with the precipitate were immediately vortex-mixed and centrifuged for 10 min at 13,000 g. One-hundred-fifty microliters of supernatant was transferred into microvials and placed in the autosampling device. Samples were analyzed using a Protecol C18HPH 150 × 4.6 mm 5 μ column (SGE Analytical Science), a double-pump HPLC apparatus (Agilent Technologies) equipped with spectrophotometric and fluorescence detectors. TRP was detected by a fluorescence detector at an excitation wavelength of 285 nm and an emission wavelength of 365 nm. Kynurenine and nitrotyrosine were detected by recording UV absorbance at a wavelength of 360 nm. Elution solvent was as follows: buffer A: potassium phosphate solution (0.015 mol/L, pH 6.4) containing 27 mL acetonitrile. Buffer B: acetonitrile. Analysis was carried out at a flow rate of 1 mL/min at 25°C for 12 min. Concentration of components was calculated according to peak heights and was compared with both 3-nitro-L-tyrosine as internal standard and reference curves constructed with L-tryptophan (concentration 10, 20, 30 mmoli/L) and KYN (10, 20, 30 mmoli/L). The intra-daily coefficient of variation was 1.20% for TRP and 1.25% for kynurenine. Inter-days coefficient was 3.5% for TRP and 3.8% for kynurenine.

### Mixed tumor-cell lymphocyte cultures (MTLC)

CD3^+^ cells were positively selected from buffy-coat preparations of volunteer blood donors with directly conjugated anti-CD3 magnetic microbeads (Miltenji). CD3^+^cells were plated on 96-well plates at 2 × 10^5^ cells/well in the supernatants obtained by MB primary cell line (#19) culture conditions. After 5 days, cells were harvested to evaluate Treg expansion. IL-2 (Immunological Science) was added to the MTLC at 10 IU/ml.

### FACS analysis

Treg expansion was evaluated following the manufacturer's protocol (BD Pharmingen). Briefy CD3+ cells, cultured in Daoy supernatants, were harvested and washed with PBS 1% BSA and stained for APC-conjugated anti CD4 and PE-conjugated and CD25 antibody for 15 minutes. Cells were then washed and re-suspended in fixation buffer for 10 minutes. Upon centrifugation, supernatant was discarded and cells were re-suspended in permeabilization buffer for 30 minutes. Upon washing of the cells, samples were stained with FITC-conjugated anti FoxP3 antibody for 30 minutes. At the end of the staining process, samples were washed and re-suspended in PBS for FACS analysis (FACS CANTO II, BD).

## SUPPLEMENTARY MATERIAL FIGURES


