# Supplementary material for: IDO1 involvement in mTOR pathway: a molecular mechanism of resistance to mTOR targeting in medulloblastoma
Source: Oncotarget. 2016 May 11;7(33):52900–11. doi: 10.18632/oncotarget.9284 (PMC5288157; doi:10.18632/oncotarget.9284)
Supplement: Supplementary file 1 [file oncotarget-07-52900-s001.pdf]

IDO1 involvement in mTOR pathway: A molecular mechanism of resistance to mTOR targeting in medulloblastoma

Supplementary Materials

A

Dataset: Robinson G et al Nature 2012 (DNA)

GEO ID: gse37418

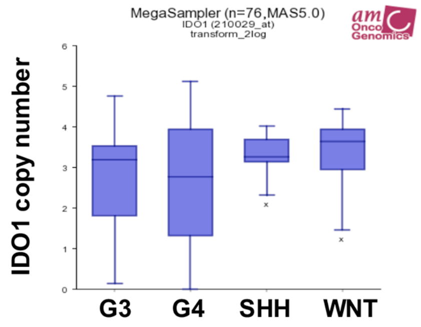

One Way Analysis of variance (ANOVA)

ANOVA	sum_square	df	mean_square	F	p-value
Between	4.313	3	1.438	0.788	0.50
Within	25.900	69	1.825	-	-

B

Dataset: Kool M et al Plos One 2008 (RNA)

GEO ID: GSE10327

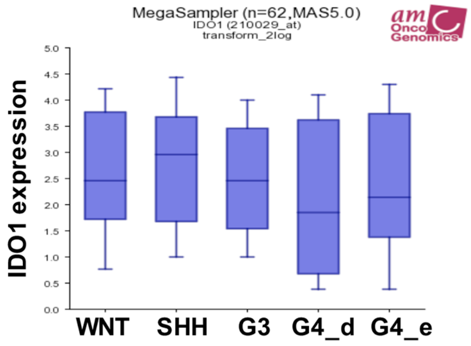

One Way Analysis of variance (ANOVA)

ANOVA	sum_square	df	mean_square	F	p-value
Between	4.331	4	1.083	0.736	0.57
Within	83.800	57	1.470	-	-

Supplementary Figure S1: IDO1 expression by Dataset analysis from R2: Genomics Analysis and Visualization Platform. (A–B) BoxPlot showing DNA profiling data (A) and mRNA expression data (B) form the indicated dataset of Medulloblastoma subdivided according to molecular subgroup. The boxes represent the 25th to 75th percentile with the median depicted as a horizontal line. Extremes are indicated by the whiskers, and the presence of outliers is indicated by (o). One Way Analysis of variance (ANOVA) across the different groups is also showed.

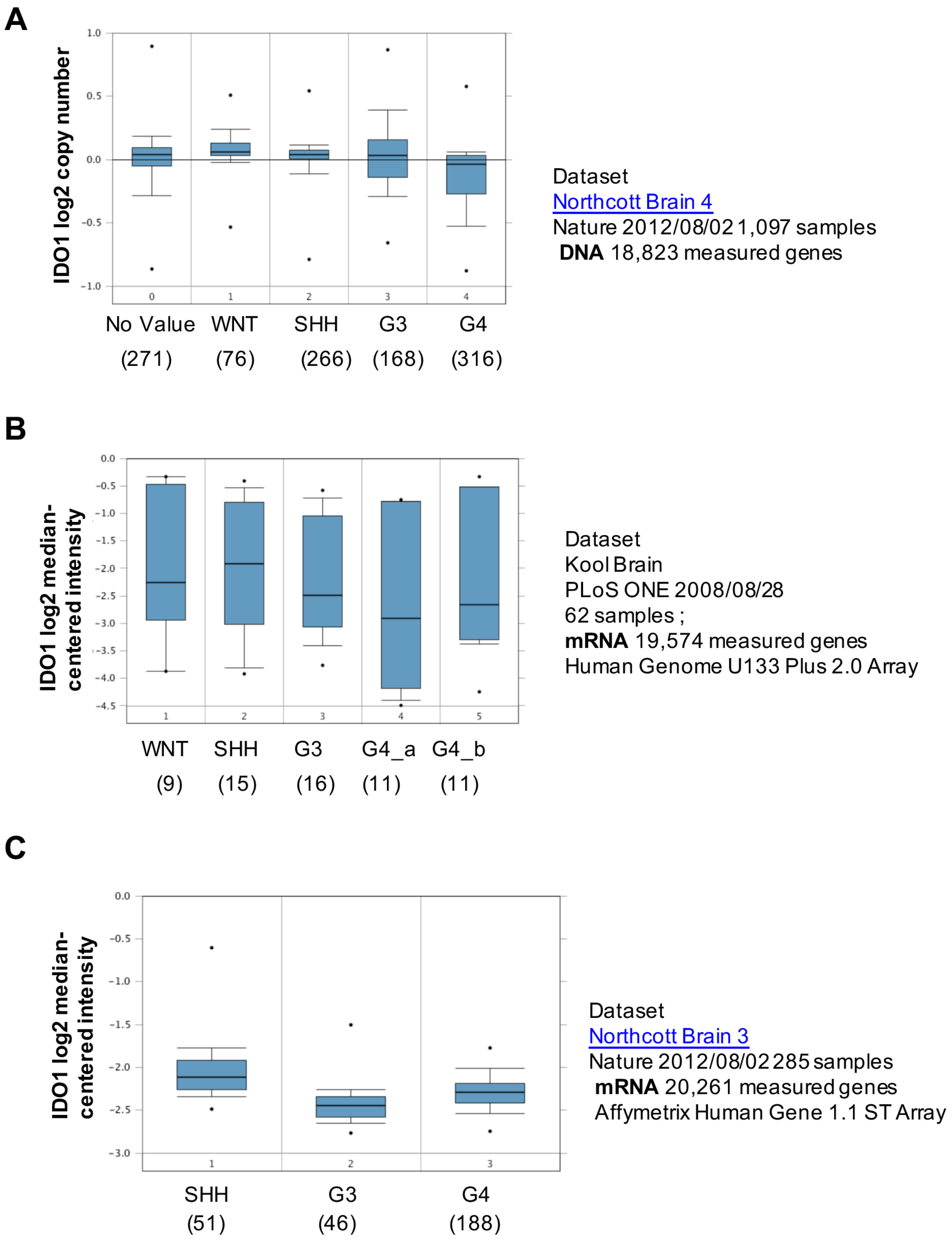

Supplementary Figure S2: IDO1 expression by Dataset analysis from Oncomine Platform. (**A–C**) BoxPlot showing DNA profiling data (A) and mRNA expression data (**B–C**) form the indicated dataset of Medulloblastoma subdivided according to molecular subgroup. The boxes represent the 25th to 75th percentile with the median depicted as a horizontal line. Extremes are indicated by the whiskers, and the presence of outliers is indicated by (o). Details on the numbers of sample analyzed, measured genes and used array are also reported.
